# MicroRNA-21 prevents excessive inflammation and cardiac dysfunction after myocardial infarction through targeting KBTBD7

**DOI:** 10.1038/s41419-018-0805-5

**Published:** 2018-07-10

**Authors:** Linshan Yang, Bo Wang, Qingqing Zhou, Yiru Wang, Xingguang Liu, Zhongmin Liu, Zhenzhen Zhan

**Affiliations:** 1 Institute of Heart Failure, Shanghai East Hospital, Tongji University School of Medicine, 200120 Shanghai, China; 2 Key Laboratory of Arrhythmias of the Ministry of Education of China, Shanghai East Hospital, Tongji University School of Medicine, 200120 Shanghai, China; 3 School of Preclinical Medicine, Guangxi Medical University, 530021 Nanning, Guangxi China; 4 Department of Anesthesiology, Changzheng Hospital, Second Military Medical University, 200433 Shanghai, China; 5 National Key Laboratory of Medical Immunology & Institute of Immunology, Second Military Medical University, 200433 Shanghai, China

## Abstract

The excessive inflammation triggered by damage-associated molecular patterns (DAMPs) after myocardial infarction (MI) is responsible for the development of cardiac dysfunction and adverse remodeling, while the mechanisms by which inflammation is fine tuned remain to be fully elucidated. MicroRNA-21 (miR-21) has been shown to function in cardiovascular diseases, while its role in inflammatory responses and cardiac function post MI in mice remains unknown. Here, we found that miR-21 expression was markedly increased in border and infarct areas of cardiac tissues during the early inflammatory phase of MI model established by ligating the left-anterior descending coronary artery. MiR-21 knockout mice had decreased survival rates, worse cardiac dysfunction, and increased infarct and scar areas after MI compared with WT mice. MiR-21 knockout mice showed significantly higher levels of inflammatory cytokines including IL-1β, IL-6, and TNF-α in cardiac tissues, as well as infiltration of CD11b^+^ monocytes/macrophages with higher expression level of inflammatory cytokines. MI induced the great release of high mobility group protein B1 (HMGB1) and heat shock protein 60 (HSP60) in cardiac tissue. MiR-21 deficiency significantly promoted the inflammatory cytokine production triggered by DAMPs in macrophages, whereas, miR-21 overexpression markedly inhibited the inflammatory cytokine production. Mechanistically, miR-21 deficiency enhanced p38 and NF-κB signaling activation in cardiac tissue post MI and macrophages treated with DAMPs. MiR-21 was found to directly target kelch repeat and BTB (POZ) domain containing 7 (KBTBD7), which promoted DAMP-triggered inflammatory responses in macrophages. Furthermore, KBTBD7 interacted with MKK3/6 and promoted their activation, which in turn enhanced the activation of downstream p38 and NF-κB signaling induced by DAMPs. Therefore, our findings demonstrate that miR-21 attenuates inflammation, cardiac dysfunction, and maladaptive remodeling post MI through targeting KBTBD7 and inhibiting p38 and NF-κB signaling activation, suggesting that miR-21 may function as a novel potential therapeutic target for MI.

## Introduction

Myocardial infarction (MI) resulting from coronary artery occlusion causes ischemic death of cardiac tissue. The pathological changes post MI in cardiac tissue can be divided into three overlapping phases: inflammatory phase, proliferative phase, and maturation phase^1,2^. In the acute inflammatory phase of MI, massive cellular necrosis and matrix degradation productions lead to the release of endogenous damage-associated molecular patterns (DAMPs) such as high mobility group box 1 (HMGB1) and heat shock proteins (HSPs)^2,3^. DAMPs can be recognized by Toll like receptors (TLRs) such as TLR4 mainly expressed on macrophages which are abundant in cardiac tissue, which in turn activates downstream MAPK and NF-κB signaling, resulting in the production of inflammatory cytokines such as IL-1β, IL-6, and TNF-α by macrophages^4,5^. Next, during the proliferative phase, macrophages phagocytose the apoptotic or necrotic cells and activate an anti-inflammatory program through the release of TGF-β and IL-10^5^. Then TGF-β induces the differentiation of myofibroblast, which promotes extracellular matrix protein deposition in infarcted area^6^. Thus, macrophage is a principle inflammatory cell type that triggers and modulates the inflammatory response after MI^5,7^. Increasing evidence indicates that the inflammatory response elicited by MI are essential for the initiation and progression of the subsequent repair response, however, the excessive inflammation has an important role in the development of adverse cardiac remodeling and the functional deterioration of heart^2,7,8^. Thus, post-infarction inflammation is a double-edged sword, and how to control inflammation to prevent pathological remodeling is an important issue, which remains to be fully elucidated.

MicroRNAs (miRNAs) are a kind of natural, endogenous, non-coding small RNAs, which bind the 3′-untranslated region of their target mRNAs and negatively regulate gene expression via mRNA degradation or translation inhibition^9^. MiRNAs are the pivotal regulators not only in normal cell development and physiological processes, but also in many pathological states^10^. It has been shown that miRNAs have crucial roles in cardiovascular development and diseases^11,12^. However, miRNAs which are involved in the inflammatory response post MI need to be further identified.

MicroRNA-21 (miR-21) has a crucial role in a variety of biological functions and diseases including development, cancer, pulmonary diseases, and autoimmune diseases by regulating some target proteins^13^. In recent years, miR-21 has been uncovered to function in cardiovascular diseases. MiR-21 promotes cardiac fibroblast survival and growth factor secretion, and then aggravates the extent of interstitial fibrosis and cardiac hypertrophy in a mouse pressure-overload-induced disease model through inhibition of sprouty homolog 1 (Spry1) in fibroblasts^14^. MiR-21 also has a protective role in cardiomyocyte apoptosis after myocardial ischemia– reperfusion (I/R) injury through the PTEN/Akt signaling pathway and suppression of FasL, as well as in H_2_O_2_-induced cardiac cell death and apoptosis via its target gene programmed cell death 4 (PDCD4)^15–17^. Although these previous studies about miR-21 have focused on its function in myocardial fibrosis and cardiomyocyte apoptosis, the role of miR-21 in inflammation and cardiac function after MI remains unclear. Here we found that miR-21 deficiency could promote inflammatory responses in cardiac tissue and macrophages through targeting KBTBD7 after MI, which reduced the survival rate and cardiac function as well as increased infarct and scar sizes. Our study provides a novel insight into the regulation of post-infarction inflammation and cardiac function via miR-21.

## Materials and methods

### MiR-21 knockout (KO) mice and mouse model of MI

MiR-21 KO mice were from Shanghai Research Center for Model Organisms (Shanghai, China) and bred in specific pathogen-free conditions. Wild-type C57BL/6 mice were obtained from Joint Ventures Sipper BK Experimental Animal Company (Shanghai, China). All animal experiments were in accordance with the National Institute of Health Guide for the Care and Use of Laboratory Animals, with the approval of the Scientific Investigation Board of Tongji University, Shanghai. Eight- to ten-week-old male littermate mice were randomly assigned to receive MI operation as described previously^18^. Briefly, mice were anesthetized with an intraperitoneal injection of a mixture of ketamine (50 μg/g body weight) and sodium pentobarbital (60 μg/g body weight), then intubated and placed on a rodent ventilator. The chest cavity was opened with a small incision (5 mm in length) at the level of the fourth and fifth intercostal space. The left-anterior descending coronary artery (LAD) was visualized under a microscope and ligated by using a 7–0 prolene suture. The sham operated mice underwent the same procedure without tying the suture but moving suture behind the LAD artery. In some experiments, adenovirus particles overexpressing miR-21 (Ad-miR-21) or silencing KBTBD7 (K7 si), or control adenovirus particles in a volume of 50 μl were intramyocardially injected into the infarct border zone using a 30-gauge needle^19^. At endpoint, mice were anesthetized and euthanized by CO_2_ asphyxiation for the collection of heart.

### Transthoracic echocardiography

Left-ventricular performance was assessed by transthoracic two-dimensional echocardiography using a Vevo 2100 high-resolution imaging system with a 30-MHz transducer (Visual Sonics, Canada) as previously used^20^. Left-ventricular internal diameter diastole (LVIDd) and left-ventricular internal diameter systole (LVIDs) were acquired from M-mode tracings from parasternal short-axis views at the mid and apical levels at each time point. Left-ventricular end-diastolic volume (LVEDV) and left-ventricular end-systolic volume (LVESV) were measured through parasternal long-axis scans. The percentages of left-ventricular ejection fraction (LVEF) and left-ventricular fractional shortening (LVFS) were calculated with corresponding formulas.

### Histological analysis

Heart tissue was fixed with 4% paraformaldehyde for 24 h, dehydrated through increasing concentrations of ethanol, and then embedded in paraffin. Masson trichrome staining was performed on heart tissue sections (5 μm). The percent of scar circumference of LV was quantified with ImageJ analysis software (National Institutes of Health). The myocardial infarct size was analyzed by triphenyltetrazolium chloride (TTC) staining as described^18^. The heart sections were also subjected to immunohistochemistry staining with antibodies against IL-1β, IL-6 and TNF-α (ebioscience), HMGB1 and HSP60 (Biosynthesis Biotechnology), and immunofluorescence analysis with antibodies against IL-1β and CD11b (ebioscience).

### Cell culture and transfection

Thioglycollate-elicited mouse peritoneal macrophages were prepared as described previously^21^. In brief, cells were harvested by peritoneal washing with serum-free DMEM from mice killed by CO_2_ asphyxiation. The pellet was resuspended in DMEM supplemented with 10% FBS (Gibco) and plated in cell culture plates. After culture for 1 h, nonadherent cells were removed by gentle washing with DMEM while adherent macrophages were maintained in culture until use. Peritoneal macrophages from WT mice were transfected with miR-21 mimics or control mimics (RiboBio) through INTERFERin reagent (Polyplus-transfection) according to the standard protocol. Recombinant mouse HMGB1 and HSP60 proteins used to stimulate macrophages were from CUSABIO, and endotoxin level was <0.01 ng/μg recombinant protein as determined by LAL assay. CD11b^+^ monocytes/macrophages in the border/infarct areas of cardiac tissues after MI or left-ventricular anterior wall from sham group mice were isolated by positive selection using CD11b microbeads and magnetic cell sorting separator (Miltenyi Biotec). Briefly, to prepare single-cell suspensions, the border/infarct area or healthy left ventricle anterior wall in the harvested hearts were excised and minced with fine scissors, then digested in a cocktail of collagenase I, collagenase XI, DNase I, and hyaluronidase (Sigma-Aldrich) at 37 °C for 1 h with gentle shake. Dissociated cell suspensions were filtered, centrifuged, and resuspended, followed by Ficoll density gradient (GE Healthcare) to acquire mononuclear cells. CD11b^+^ monocytes/macrophages were separated by positive selection using CD11b microbeads from the mononuclear cells.

### RNA interference

The sequence of small interfering RNA (siRNA) targeting *Kbtbd7* was 5′-GGAUUAAUAUAGGCACCAU-3′. The sequence of control small RNA was 5′-UUCUCCGAACGUGUCACGU-3′. SiRNAs were synthesized by Genepharma (Shanghai, China). The siRNA duplexes were transfected into mouse peritoneal macrophages using INTERFERin (Polyplus-transfection) according to the standard protocol and as described previously^21^.

### Real-time quantitative PCR

Total RNA was extracted with TRIzol reagent (Invitrogen) and then subjected to reverse-transcription reaction using the First Strand cDNA Synthesis kit (Toyobo). cDNA was used to real-time quantitative PCR (Q-PCR) analysis with Applied Biosystems 7500 System (ThermoFisher). Data were normalized by the level of *U6*, *Gapdh*, or *β-actin* expression in each sample. Q-PCR primers were shown in Supplementary Table S1.

### Immunoblot and immunoprecipitation analysis

Cells or tissues were lysed with cell lysis buffer (Cell signaling Technology) supplemented with a protease inhibitor ‘cocktail’ (Merk), then protein concentrations in the extracts were measured by BCA assay (ThermoFisher). Immunoprecipitation (200 μg protein for one group) and immunoblot (40–50 μg protein for one gel lane) analysis were performed as described^21,22^. Antibodies to phosphorylated and total proteins of ERK, JNK, p38, p65, IKKα/β, MKK3, and MKK6 were from Cell Signaling Technology. Antibodies to GAPDH and β-actin were from Cell Signaling Technology. Antibody to KBTBD7 was from Novus Biologicals.

### 3′-UTR luciferase reporter assay

Mouse KBTBD7 mRNA 3′-UTR harboring the predicted miR-21 binding site was amplified and cloned into pGL3-promoter vector (Promega) to generate wild-type and mutant KBTBD7 3′-UTR luciferase reporter constructs. The luciferase reporter constructs and miR-21 mimics were co-transfected into HEK293 cells (American Type Culture Collection) using JetPRIME transfection reagents (Polyplus-transfection). Twenty-four hours after transfection, luciferase activity was measured by using the Dual-Luciferase Reporter Assay System (Promega)^21^.

### Statistical analysis

Data are presented as mean ± SEM. Statistical difference was determined by an unpaired two-tailed Student’s *t*-test, one-way ANOVA, or two-way ANOVA. Survival was analyzed by the Kaplan–Meier method, and differences between groups were determined by log‐rank (Mantel–Cox) test. Differences with *P* < 0.05 were considered statistically significant.

## Results

### MiR-21 deficiency promotes inflammatory responses in heart post MI

**Fig. 1:**
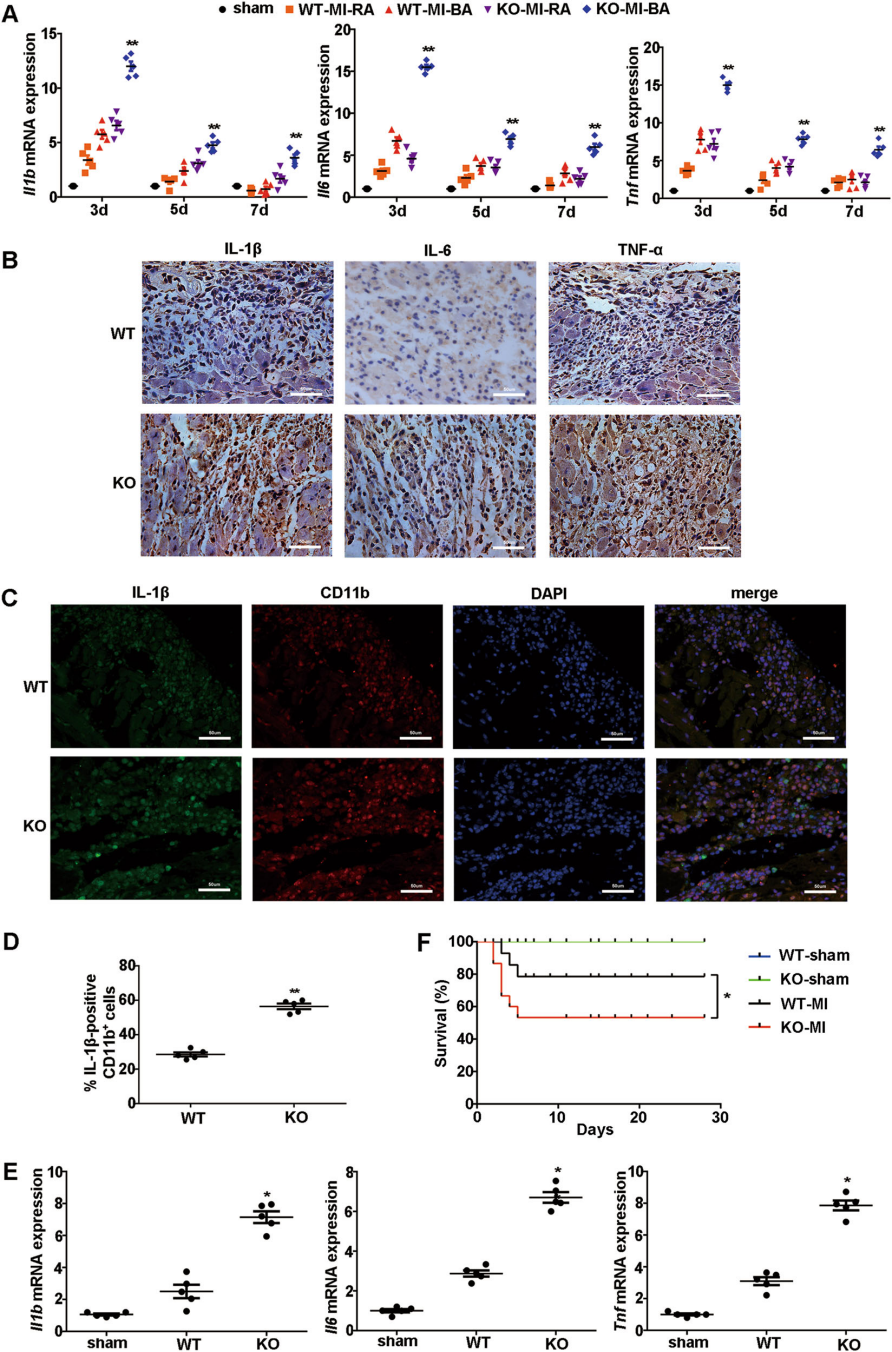
MiR-21 deficiency exacerbates inflammatory responses in heart after MI. **a** Q-PCR analysis of *Il1b*, *Il6*, and *Tnf* mRNA in heart tissues from sham group or remote area (RA) or border area (BA) from miR-21 knockout (KO) or wild-type (WT) mice at the indicated days after MI. Expression levels were presented as relative fold values compared to sham group (determined as 1 for each gene at the indicated days) after normalization to GAPDH. Data represent the mean ± SEM (*n* = 5 mice per group). **P *<* 0.01 vs. corresponding WT-MI-BA (two-way ANOVA). **b** Immunohistochemical staining of IL-1β, IL-6, and TNF-α expression in heart sections of border areas from miR-21 KO or WT mice at day 3 after MI. Scale bars = 50 μm. **c** Immunofluorescence staining of IL-1β (green) and CD11b (red) in heart sections of border areas from miR-21 KO or WT mice at day 3 after MI. Scale bars = 50 μm. **d** Quantification of IL-1β-positive cells was presented as a percentage of total CD11b^+^ cells counted. Data represent the mean ± SEM (*n* = 5 mice per group). **P < 0.01 vs. WT (Student’s *t*-test). **e** Q-PCR analysis of *Il1b*, *Il6*, and *Tnf* mRNA in CD11b^+^ monocytes/macrophages isolated from the border/infarct areas of cardiac tissues of miR-21 KO and WT mice at day 3 after MI or left-ventricular anterior wall from sham group mice. Expression levels were presented as relative fold values compared to sham group (determined as 1 for each gene) after normalization to GAPDH. Data represent the mean ± SEM (*n* = 5 mice per group). *P *<* 0.05 vs. WT (two-way ANOVA). **f** Survival curve of miR-21 KO or WT mice subjected to MI or sham operation (*n* = 16 mice per group). *P *<* 0.05 vs. WT-MI (log‐rank test)

We first investigated the expression pattern of miR-21 in cardiac tissue post MI. MiR-21 expression was significantly increased at day 1, peaked at day 3, and still remained at high levels at day 5 and day 7 after MI in infarcted and ischemic cardiac tissue (Fig. S1A). To elucidate the role of miR-21 in post-infarction inflammation, miR-21 knockout mice with miR-21 deficiency in cardiac tissue and wild-type mice were used to construct MI models (Fig. S2). MiR-21 deficiency increased cardiomyocyte apoptosis in cardiac tissue post MI (Fig. S3), which was consistent with the previous study^15^. MiR-21 knockout mice showed significantly higher levels of inflammatory cytokines including IL-1β, IL-6, and TNF-α in the border area of cardiac tissue than wild-type mice did after MI (Fig. 1a, b). Since inflammatory cytokines are predominantly produced by monocytes and macrophages in cardiac tissue following MI^3,5,7^, and the highest increased level of miR-21 was showed in CD11b^+^ monocytes/macrophages as compared with that in cardiomyocytes or fibroblasts isolated from cardiac tissue of sham or MI group (Fig. S1B), we next explored the effects of miR-21 deficiency on inflammatory cytokine production in these two types of immune cells. The severe infiltration of CD11b^+^ cells (monocytes and macrophages) with higher expression level of IL-1β was observed in cardiac tissues of miR-21 knockout mice after MI (Fig. 1c, d). CD11b^+^ monocytes/macrophages isolated from the infarcted and ischemic cardiac tissues of miR-21 knockout mice also had higher level of inflammatory cytokines (Fig. 1e). Furthermore, miR-21-deficient mice had higher mortality than that of wild-type mice after MI (Fig. 1f). These data indicate that miR-21 inhibits the inflammatory cytokine production in monocytes/macrophages of cardiac tissue, leading to the attenuated inflammatory responses in heart post MI.

### MiR-21 inhibits DAMP-triggered inflammatory cytokine production in macrophages

**Fig. 2:**
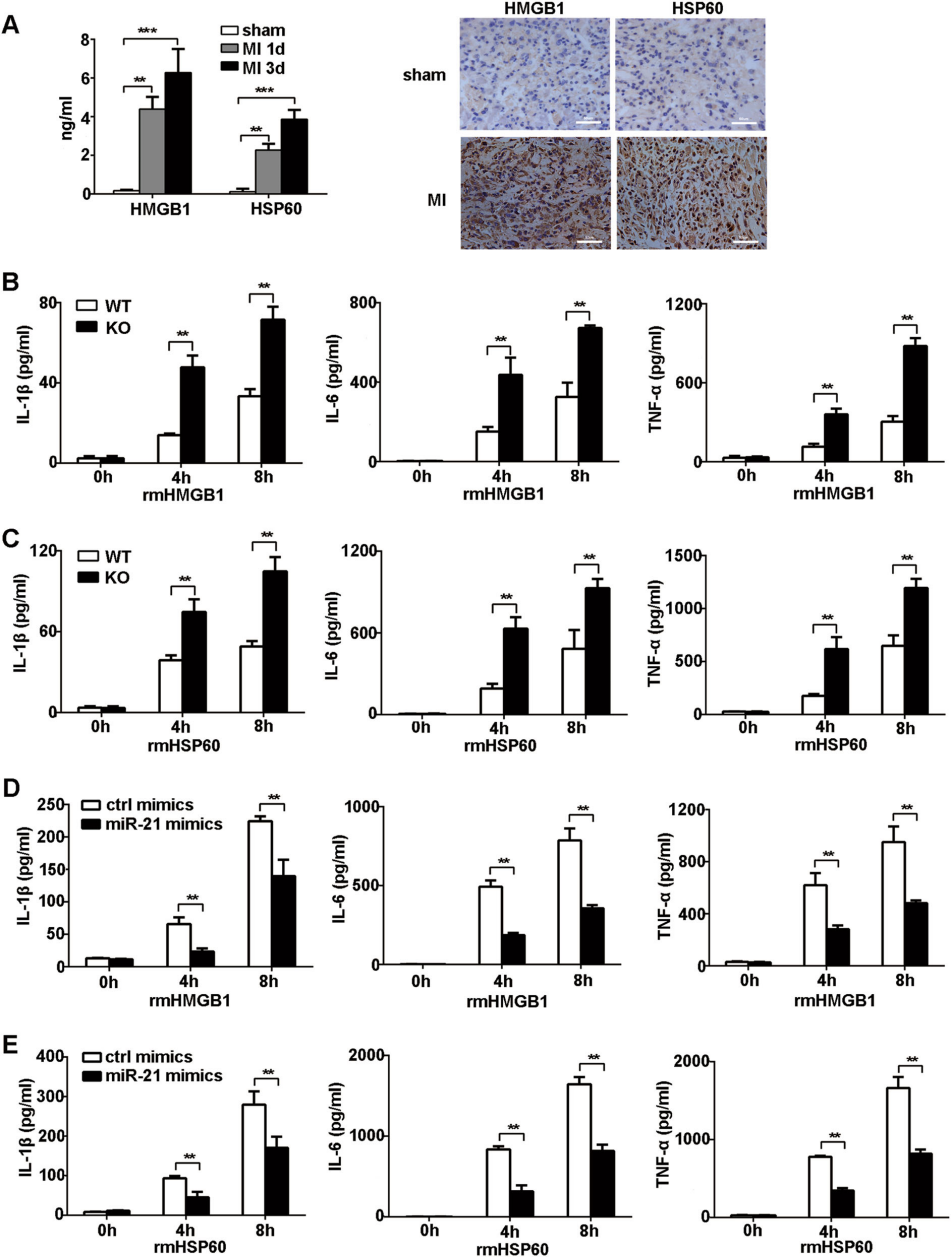
MiR-21 inhibits DAMP-triggered inflammatory cytokine production. **a** HMGB1 and HSP60 production in homogenate of heart tissues (left) or sections (right) in WT mice at the indicated days after MI or sham operation was, respectively, detected by ELISA or immunohistochemical staining. Scale bars = 50 μm. **b**, **c** ELISA of cytokines in supernatants of peritoneal macrophages isolated from miR-21 knockout (KO) or littermate control WT mice stimulated with rmHMGB1 (1 μg/ml) (**b**) or rmHSP60 (1 μg/ml) (**c**) for the indicated times. Data represent the mean ± SEM (*n* = 6 independent preparations). **d**, **e** ELISA of cytokines in supernatants of macrophages 48 h after transfection with miR-21 or control mimics followed by treatment with rmHMGB1 (1 μg/ml) (**d**) or rmHSP60 (1 μg/ml) (**e**) for the indicated times. Data represent the mean ± SEM (*n* = 6 independent preparations). **P *<* 0.01, ***P *<* 0.001 (two-way ANOVA)

DAMPs released after MI can activate TLRs mainly expressed in macrophages and initiate inflammatory responses^3, 4^. Then we examined the effects of miR-21 on DAMP-triggered inflammatory cytokine production in macrophages. The significant increase of HMGB1 and HSP60 release was observed in cardiac tissue at day 1 or day 3 after MI (Fig. 2a). Furthermore, recombinant mouse HMGB1 (rmHMGB1) or HSP60 (rmHSP60) treatment significantly upregulated miR-21 expression in a time-dependent manner in macrophages (Fig. S4). The stimulation of macrophages with rmHMGB1 or rmHSP60 resulted in a dose-dependent increase in IL-6 production levels (Fig. S5). So, 1 μg/ml of rmHMGB1 or rmHSP60 was used to treat macrophages and found that miR-21 deficiency markedly promoted the production of IL-1β, IL-6, and TNF-α at the mRNA and protein levels in macrophages (Fig. 2b, c; Fig. S6). Accordingly, overexpression of miR-21 mimics significantly downregulated the mRNA and protein expression levels of IL-1β, IL-6, and TNF-α in wild-type macrophages stimulated with rmHMGB1 or rmHSP60 (Fig. 2d, e; Fig. S7). These results indicate that miR-21 acts as a negative regulator of DAMP-triggered inflammatory responses.

### MiR-21 suppresses p38 and NF-κB signaling activation post MI

**Fig. 3:**
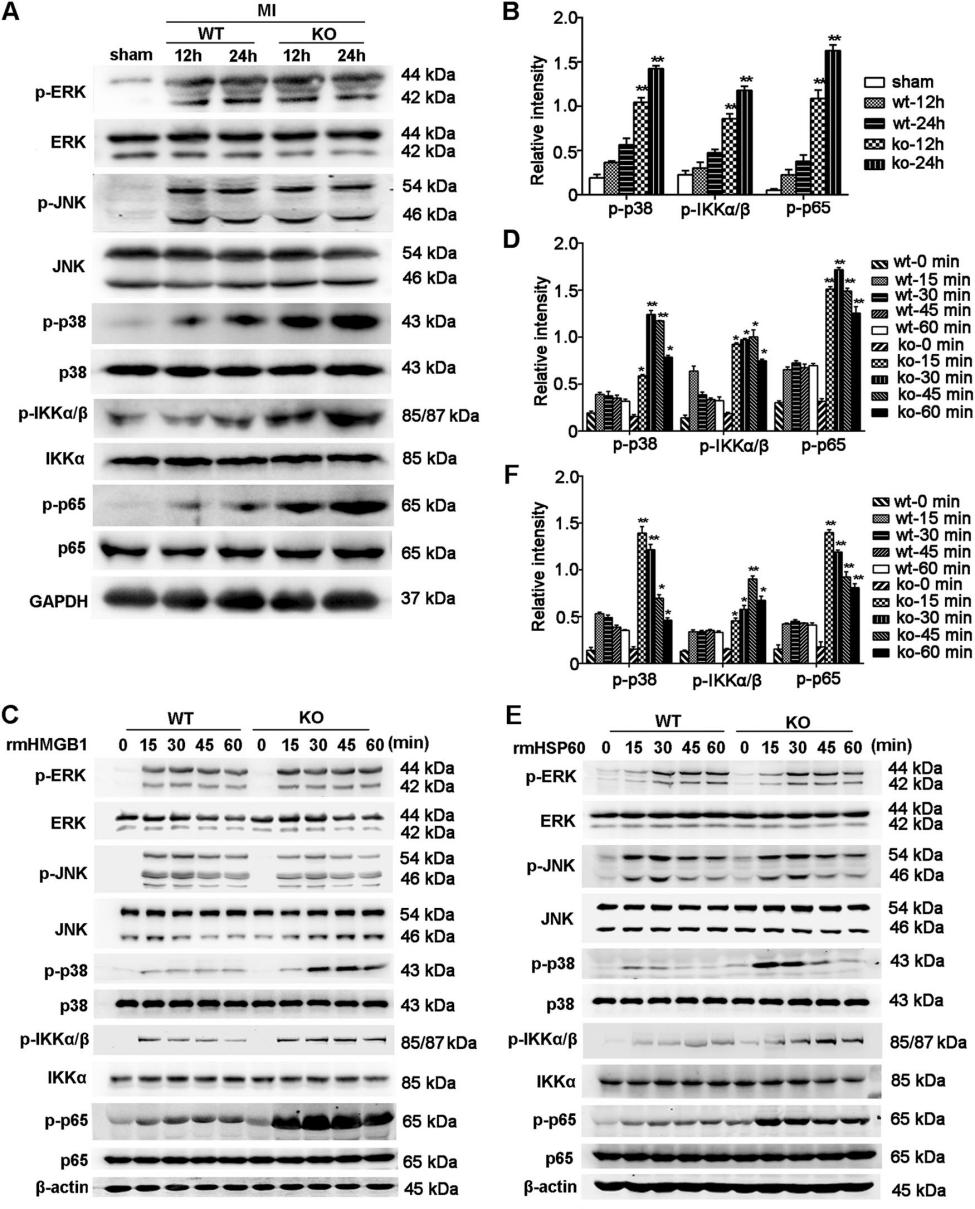
MiR-21 suppresses DAMP-induced activation of p38 and NF-κB signaling. **a**, **b** Immunoblot analysis of phosphorylation (p-) levels of proteins in lysates of heart tissues from sham group or miR-21 KO and WT mice at the indicated times after MI. **c**–**f** Immunoblot analysis of phosphorylation (p-) levels of protein in lysates of miR-21-deficient or WT macrophages treated with rmHMGB1 (1 μg/ml) (**c**, **d**) or rmHSP60 (1 μg/ml) (**e**, **f**) for the indicated times. Quantification presented as the relative densitometry intensity of the phosphorylated protein to total protein in the corresponding same lane (**b**, **d**, **f**). Data represent three individual experiments and the mean ± SD. *P *<* 0.05, **P *<* 0.01 vs. WT at the same indicated times (two-way ANOVA)

Since the activation of MAPK and NF-κB signaling pathway has a critical role in the production of inflammatory cytokines induced by TLRs, which recognize DAMPs released after MI^3,4^, we further explored the effects of miR-21 on MAPK and NF-κB signaling activation. As shown in Fig. 3a, b, the phosphorylation levels of p38, IKKα/β, and p65 but not ERK or JNK were substantially upregulated in cardiac tissues from miR-21 knockout mice post MI. Moreover, miR-21 deficiency also markedly increased the phosphorylation levels of p38, IKKα/β, and p65 but not ERK or JNK induced by recombinant HMGB1 or HSP60 in macrophages (Fig. 3c–f), suggesting that miR-21 inhibits the production of inflammatory cytokines initiated by MI mainly by suppressing the activation of p38 and NF-κB signaling.

### MiR-21 directly targets KBTBD7

**Fig. 4:**
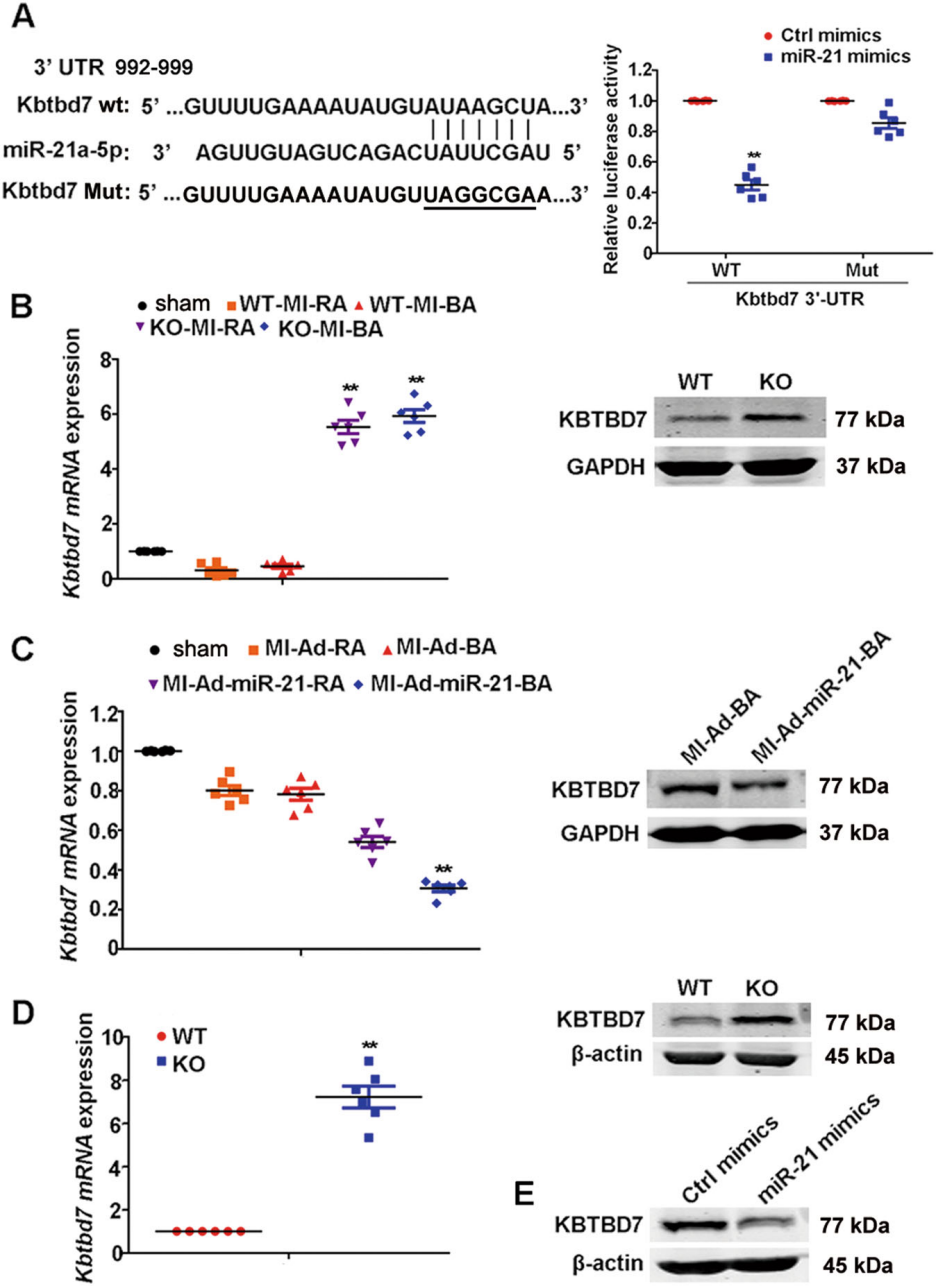
MiR-21 targets KBTBD7. **a** Shown is the alignment of miR-21, its target sites and mutant in 3′-UTR of KBTBD7 (left). Luciferase activity assay in lysates of HEK293 cells 24 h after transfection with wild-type or mutant KBTBD7 3′-UTR firefly luciferase reporter plasmids, together with miR-21 or control mimics. The firefly luciferase activity was normalized by renilla luciferase activity (Right). Data represent the mean ± SEM (*n* = 6 independent preparations). **P *<* 0.01 vs. ctrl mimics (two-way ANOVA). **b** Q-PCR analysis of *Kbtbd7* mRNA (normalization to GAPDH) or immunoblot analysis of its protein expression in heart tissues of remote area (RA) or border area (BA) from miR-21 KO or WT mice 3 days after MI. Data represent the mean ± SEM (*n* = 6 mice per group). **P < 0.01 vs. WT-MI-RA (two-way ANOVA). **c** Q-PCR analysis of *Kbtbd7* mRNA (normalization to GAPDH) or immunoblot analysis of its protein expression in heart tissues of remote area (RA) or border area (BA) from mice 3 days after MI followed by injection with miR-21 (Ad-miR-21) or control adenovirus (Ad) into peri-infarct heart tissue. Data represent the mean ± SEM (*n* = 6 mice per group). **P < 0.01 vs. MI-Ad-BA (two-way ANOVA). **d** Q-PCR analysis of *Kbtbd7* mRNA (normalization to β-actin) or immunoblot analysis of its protein expression in miR-21-deficient or WT macrophages. Data represent the mean ± SEM (*n* = 6 independent preparations). **P *<* 0.01 vs. WT (Student’s *t*-test). **e** Immunoblot analysis of KBTBD7 protein expression in macrophages 48 h after transfection with miR-21 or control mimics

Next, we investigated the target of miR-21, which might modulate DAMP-triggered inflammatory responses post MI. By prediction of new targets of miR-21 via TargetScan 7.1 (http://www.targetscan.org), we found that the 3′-UTR of mouse Kelch repeat and BTB (POZ) domain containing 7 (KBTBD7) contained a putative miR-21 target site (Fig. 4a, left). Sequence analysis indicated that this target sequence was conserved across different species including human, mouse, chimpanzee, rhesus, rat, rabbit and pig genomes (data not shown), suggesting conservational functions of this sequence. To determine whether KBTBD7 was a bona fide target of miR-21, the fragment of KBTBD7 3′-UTR containing the wild-type target sequence or the fragment whose target site was mutated, was integrated into the luciferase reporter vectors (Fig. 4a, left). By co-transfection of the reporter plasmids and miR-21 mimics in HEK293 cells, we found that miR-21 mimic markedly decreased the luciferase activity of wild-type KBTBD7 3′-UTR luciferase but had no effect on mutant KBTBD7 3′-UTR luciferase (Fig. 4a, right). Furthermore, the expression of KBTBD7 at mRNA and protein levels was much higher in border area of infarct heart from miR-21 knockout mice than that from wild-type mice (Fig. 4b). Consistently, overexpression of miR-21 in vivo through directly injecting miR-21 adenovirus (Ad-miR-21) into peri-infarct heart tissue decreased the mRNA and protein expression of KBTBD7 in the border area of infarct heart (Fig. 4c; Fig. S8). MiR-21 deficiency also upregulated the mRNA and protein expression of KBTBD7 in macrophages, and transfection of miR-21 mimics in wild-type macrophages resulted in the decreased expression of KBTBD7 (Fig. 4d, e). Together, these results reveal that miR-21 selectively targets KBTBD7 and downregulates KBTBD7 expression through both mRNA degradation and translational inhibition.

### KBTBD7 promotes DAMP-triggered inflammatory responses in macrophages

**Fig. 5:**
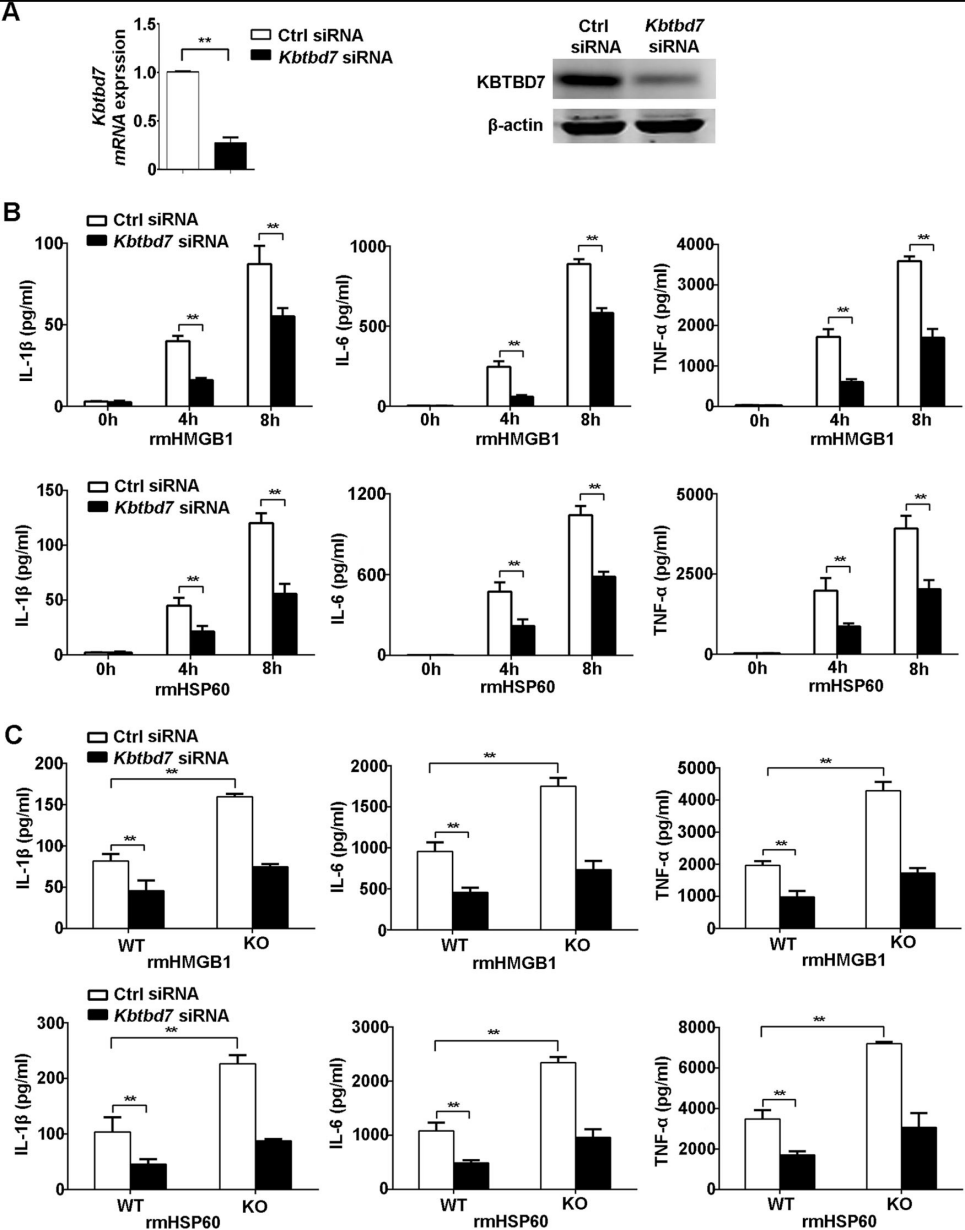
Knockdown of KBTBD7 decreases DAMP-triggered inflammatory cytokine production. **a** Q-PCR analysis of *Kbtbd7* mRNA (normalization to β-actin) or immunoblot analysis of its protein expression in macrophages transfected with control siRNA or *Kbtbd7* siRNA. **b** ELISA of cytokines in supernatants of macrophages 48 h after transfection with control siRNA or *Kbtbd7* siRNA followed by treatment with rmHMGB1 (1 μg/ml) or rmHSP60 (1 μg/ml) for the indicated times. **c** ELISA of cytokines in supernatants of miR-21-deficient or WT macrophages 48 h after transfection with control siRNA or *Kbtbd7* siRNA followed by treatment with rmHMGB1 (1 μg/ml) or rmHSP60 (1 μg/ml) for 8 h. Data represent the mean ± SEM (*n* = 6 independent preparations). **P *<* 0.01. One-way ANOVA (**a**) or two-way ANOVA (**b**, **c**)

We further observed the effect of KBTBD7 on DAMP-induced production of inflammatory cytokines in macrophages. The endogenous expression of KBTBD7 in macrophages was decreased considerably by KBTBD7-specific siRNA (Fig. 5a). KBTBD7 silencing significantly decreased the production of IL-1β, IL-6, and TNF-α at the mRNA and protein level in macrophages treated with rmHMGB1 or rmHSP60 (Fig. 5b; Fig. S9). Moreover, KBTBD7 silencing could markedly reverse the increased production of inflammatory cytokines triggered by rmHMGB1 or rmHSP60 in macrophages from miR-21-deficient mice (Fig. 5c). These data demonstrate that KBTBD7 promotes DAMP-triggered inflammatory responses, while miR-21 inhibits inflammation by targeting KBTBD7.

### KBTBD7 enhances the activation of p38 and NF-κB signaling by facilitating MKK3/6 activation

**Fig. 6:**
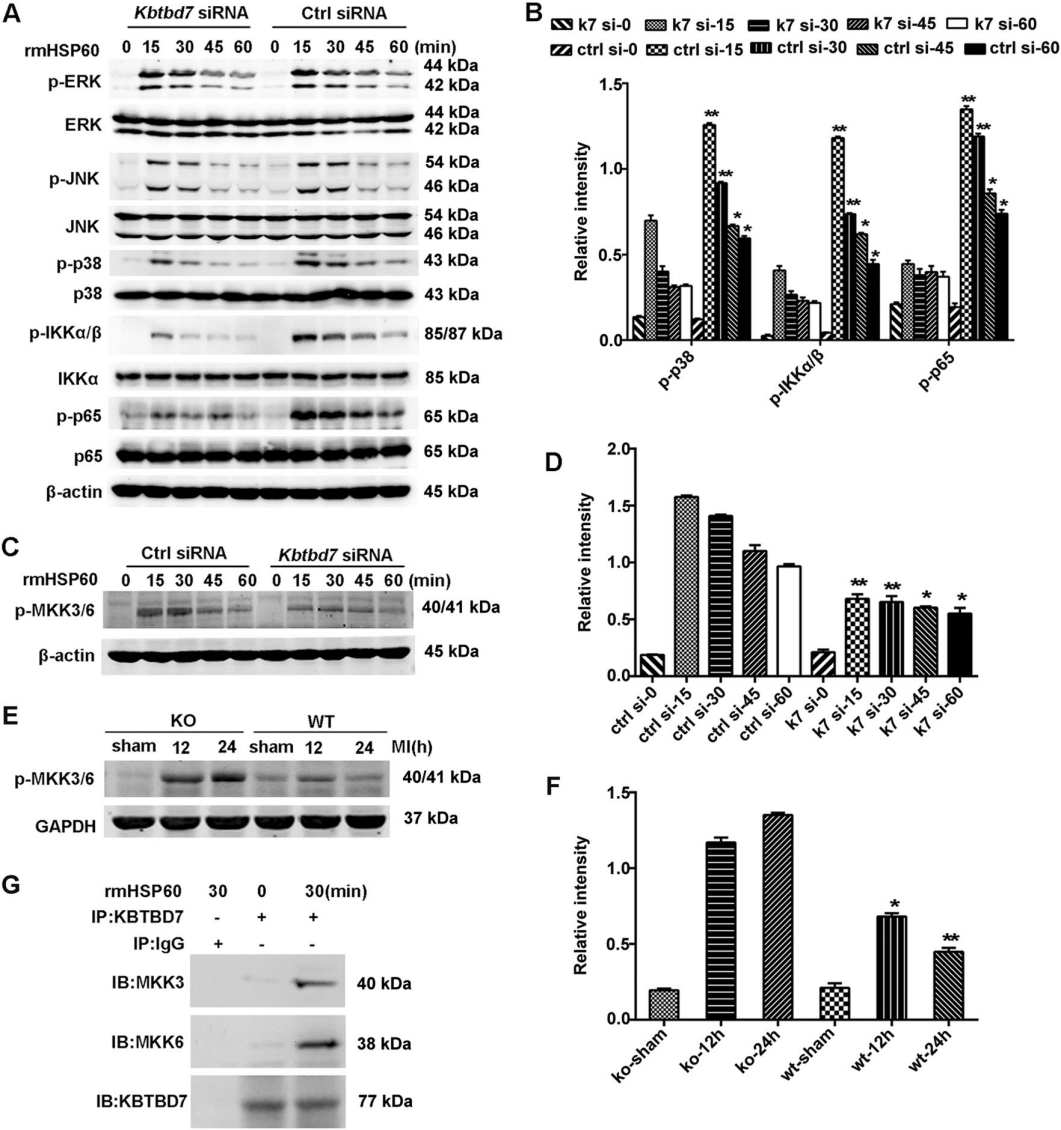
KBTBD7 enhances p38 and NF-κB activation by interacting with MKK3/6. **a**–**d** Immunoblot analysis of phosphorylation (p-) levels of proteins in lysates of macrophages 48 h after transfection with control siRNA or *Kbtbd7* siRNA followed by treatment with rmHSP60 (1 μg/ml) for the indicated times. **e**, **f** Immunoblot analysis of phosphorylation (p-) level of MKK3/6 in lysates of heart tissues from sham group or miR-21 KO and WT mice at the indicated times after MI. **g** Immunoblot analysis of MKK3, MKK6, and KBTBD7 immunoprecipitated with anti-KBTBD7 antibody from lysates of macrophages treated with rmHSP60 (1 μg/ml) for the indicated times. Quantification presented as the relative densitometry intensity of the phosphorylated protein to total protein (**b**), β-actin (**d**), or GAPDH (**f**) in the corresponding same lane. Data represent three individual experiments and the mean ± SD. *P *<* 0.05, **P *<* 0.01 vs. *Kbtbd7* siRNA (**b**), control siRNA (**d**), or KO (**f**) at the same indicated times (two-way ANOVA)

We next investigate the mechanism by which KBTBD7 enhances DAMP-triggered inflammatory responses. As shown in Fig. 6a, b, KBTBD7 silencing substantially impaired the phosphorylation of p38, IKKα/β, and p65 but not ERK and JNK in macrophages stimulated with rmHSP60, suggesting that KBTBD7 promotes the activation of p38 and NF-κB signaling induced by DAMPs, which is consistent with the enhanced activation of p38 and NF-κB signaling in cardiac tissues and macrophages from miR-21-deficient mice (Fig. 3). Since previous studies show that MAP kinase kinases 3/6 (MKK3/6) function as proximal-activating kinases in the p38 MAPK pathway and also activate the NF-κB pathway^23,24^, we further determined whether KBTBD7 silencing or miR-21 deficiency affected the phosphorylation of MKK3/6. As shown in Fig. 6c, f, the phosphorylation of MKK3/6 was markedly downregulated in KBTBD7-silenced macrophages with rmHSP60 stimulation, whereas upregulated in cardiac tissues from miR-21 knockout mice post MI. Furthermore, KBTBD7 could interact with MKK3 and MKK6 in macrophages upon rmHSP60 stimulation (Fig. 6g). These results demonstrate that KBTBD7 interacts with MKK3/6 and promotes their activation, which in turn enhances the activation of downstream p38 and NF-κB signaling and facilitates the inflammatory cytokine production induced by DAMPs in macrophages.

### MiR-21 deficiency aggravates cardiac dysfunction after MI

**Fig. 7:**
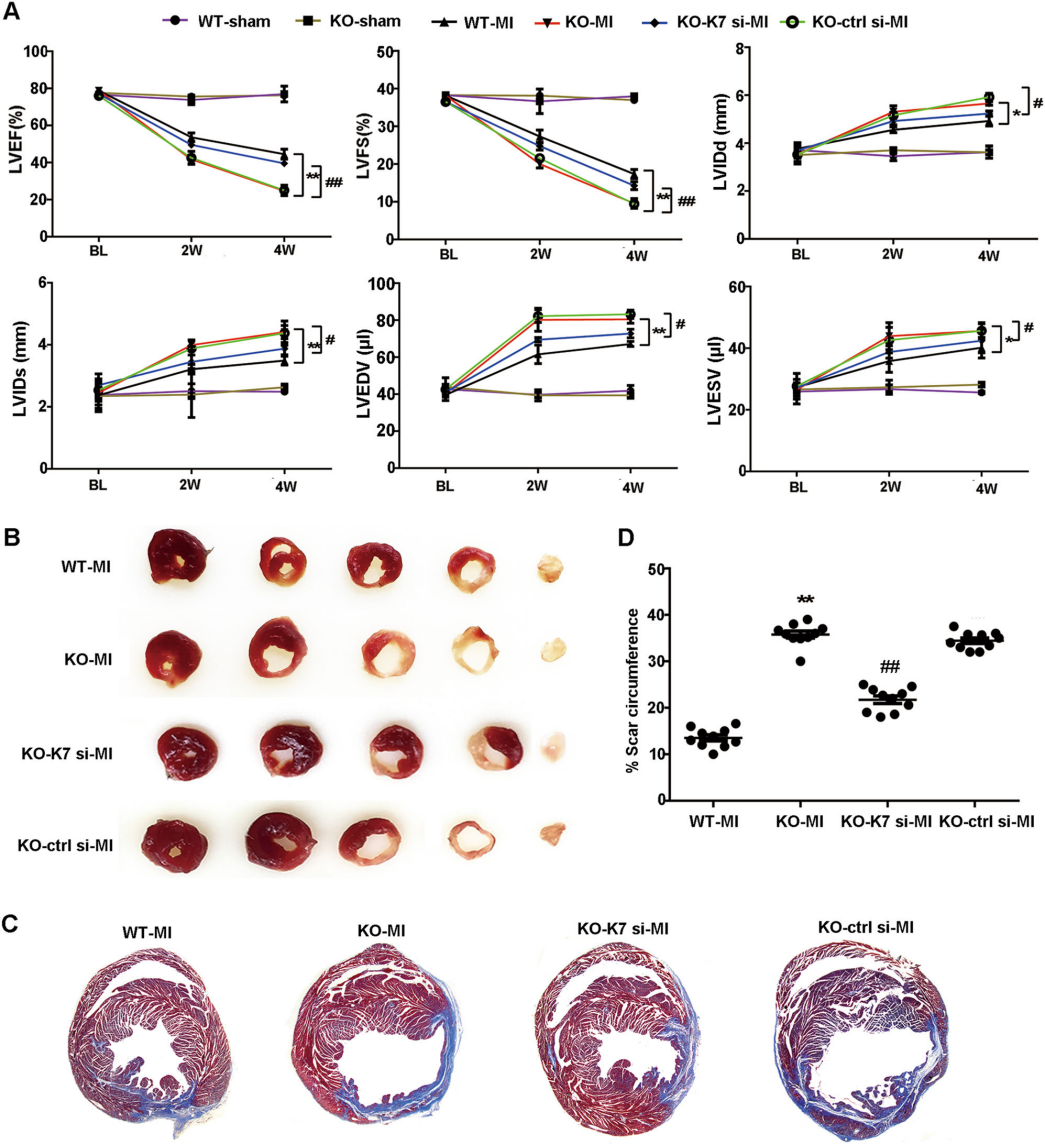
MiR-21 deficiency aggravated cardiac dysfunction post MI. **a** MiR-21 WT or KO mice underwent baseline (BL) transthoracic echocardiographic examination. Mice were then subjected to MI or sham operation, or KO mice received MI were further injected with adenovirus expressing *Kbtbd7* siRNA (KO-K7 si-MI) or control siRNA (KO-ctrl si-MI) into peri-infarct heart tissue. Mice were then followed with serial echocardiography at the indicated time points. LVEF, LVFS, LVIDd, LVIDs, LVEDV, and LVESV at different time points were shown. Data represent the mean ± SEM (*n* = 10 mice per group). *P *<* 0.05, **P *<* 0.01 WT-MI vs. KO-MI; ^#^*P* *<* 0.05, ^##^*P* *<* 0.01 KO-K7 si-MI vs. KO-ctrl si-MI (two-way ANOVA). **b** Representative images were triphenyltetrazolium chloride (TTC)-stained heart sections from miR-21 WT or KO mice 1 week after treatment as in (**a**) showing infarct zone (white area). **c**, **d** Representative images of heart sections from miR-21 WT or KO mice stained with Masson trichrome at 4 weeks after treatment as in **a**, and scar circumference was measured and expressed as a percentage of total area of left-ventricular myocardium. Data represent the mean ± SEM (*n* = 10 mice per group). **P *<* 0.01 vs. WT-MI; ^##^*P* *<* 0.01 vs. KO-ctrl si-MI (two-way ANOVA)

Finally, we examined the effect of miR-21 deficiency on cardiac function and tissue injury post MI. MiR-21-deficient mice had worse cardiac function as showed by the significantly decreased LVEF and LVFS but increased LVIDd, LVIDs, LVEDV, and LVESV as compared with those of wild-type mice (Fig. 7a; Fig. S10). However, KBTBD7 silencing in vivo by injecting adenovirus expressing KBTBD7-specific siRNA into peri-infarct areas of heart tissues of miR-21-deficient mice restored the impaired cardiac function (Fig. 7a; Fig. S11). The myocardial infarct sizes at day 7 and scar tissue percent circumferences at day 28 after MI were significantly increased in miR-21-deficient mice than those in wild-type mice, whereas KBTBD7 silencing in vivo reduced the myocardial infarct and scar sizes in miR-21-deficient mice (Fig. 7b–d; Fig. S12). Collectively, these data indicate that miR-21 deficiency aggravates cardiac dysfunction and myocardial infarct after MI, which attributes to the enhanced inflammatory response mediated by the interaction of target molecule KBTBD7 and MKK3/6.

## Discussion

The innate immune and inflammation response has been implicated in the pathogenesis of MI, orchestrating beneficial homeostatic responses in the heart following MI for cardiac repair. Inappropriate inflammatory responses lead to function deterioration and maladaptive remodeling of heart^2,8^. Macrophages are among the largest cardiac resident cell populations and have dominated roles in the inflammatory response in cardiac tissue post MI^5,25^. Herein, we demonstrate that miR-21 deficiency can facilitate inflammation in cardiac tissue and inflammatory cytokine production in macrophages triggered by DAMPs, thus exacerbate cardiac dysfunction and increase infarct and scar sizes post MI, which is attributed to the enhanced activation of p38 and NF-κB signaling mediated by the interaction between KBTBD7 and MKK3/6. Our study identifies miR-21 as a novel negative regulator in inflammatory responses and subsequent pathological process post MI. Although enhanced cardiomyocyte apoptosis in the absence of miR-21 may accentuate inflammation, the increased production of inflammatory cytokines by macrophages is the major factor which contributes to the enhanced inflammation in miR-21-deficient cardiac tissue. Since other types of cells in cardiac tissue, such as dendritic cells, also express TLRs and can recognize DAMPs to produce inflammatory cytokines^3^, miR-21 deficiency may also affect inflammation in dendritic cells post MI. Together with the protected role of miR-21 in cardiomyocyte apoptosis and its inhibitory function in inflammation after myocardial ischemia, miR-21 may be a novel target of the drug development for the treatment of MI.

The inflammatory response initiated by the innate immune system has a crucial role in the cardiac pathological process post MI. Up to now, several miRNAs have been found to have roles in regulation of the inflammatory response after MI^26^. MiR-155 can aggravate the inflammatory response, leukocyte infiltration and tissue damage in ischemia–reperfusion injury by downregulating the expression of suppressor of cytokine signaling 1 (SOCS-1) post MI^27^. Although the expression of miR-146a/b and miR-223 are increased in cardiomyocytes after MI and cardiac ischemia, and miR-146a/b and miR-223 can negatively regulate inflammation in innate immune cells, there are still few direct evidence to confirm that miR-146a/b and miR-223 are involved in inflammation post MI^28^. Thus, the roles of miRNAs in cardiac function and inflammation after MI remain not fully clear. In our study, we provide the evidence that miR-21 serves as an important negative regulator to prevent excessive inflammation and cardiac dysfunction.

KBTBD7 is a BTB-kelch family member that contains a BTB domain at the N terminus and five kelch repeats at the C terminus. In most members of BTB family proteins, the BTB domain acts as a protein–protein interaction module that is able to both self-associate and interact with non-BTB proteins^29^. It is reported that KBTBD6/7 can mediate ubiquitylation and subsequent proteasomal degradation of T-lymphoma and metastasis gene 1 (TIAM1) through binding cullin 3 (CUL3) to form a heterodimeric Cullin-RING ligases comprising CUL3 (CRL3) complex^30^. KBTBD7 directly or indirectly positively regulates the MAPK signaling pathway through activating SRE and AP-1-mediated transcriptional activation^31^. However, the role of KBTBD7 in cardiovascular diseases remains unclear. Our findings revealed that KBTBD7, as the target gene of miR-21, promoted activation of p38 and NF-κB signaling via interacting with MKK3/6. We further found that KBTBD7 silencing impaired the phosphorylation of MKK3/6 triggered by DAMPs. Since TGF-b-activated kinase-1 (TAK1) is the upstream kinase of MKK3/6 and can activate the activation of MKK3/6^32^, it is possible that KBTBD7 enhances the interaction of TAK1 and MKK3/6, then in turn promotes the phosphorylation and activation of MKK3/6, which needs to be further investigated.

It is known that MKK3 and MKK6 are the upstream kinases that mediate the phosphorylation and activation of p38^33^. However, accumulating evidence approves that MKK3 and MKK6 can regulate NF-κB signaling activation through different mechanism in various conditions. MKK3 is reported to influence the phosphorylation of IKKα/β through the mitochondrial redox status, leading to activate NF-κB transcriptional activation and inflammatory gene expression^34^. MKK3 can activate mitogen- and stress-activated protein kinase-1 (MSK1), which functions as a nuclear kinase for p65 and phosphorylates p65 at Ser276, together with phosphorylation of nucleosome components such as histone H3, induces a distinct model of NF-κB activation^35^. MKK3 and MKK6 are responsible for the activation of p38 which leads to NF-κB transcriptional activation, resulting in inflammatory gene expression in macrophages in response to chlamydial heat shock protein^36^. These above studies are consistent with our findings that miR-21 deficiency enhances MKK3/MKK6 phosphorylation and potentiates the activation of p38 and NF-κB signaling.

In conclusion, our study demonstrates that miR-21 inhibits inflammatory responses in the early phase of MI by targeting KBTBD7 and impairing MKK3/6 activation in immune cells, which subsequently prevents excessive scar formation and improves cardiac function.

## Electronic supplementary material

Supplementary Figure andTable
