# Supplementary material for: MicroRNA-21 prevents excessive inflammation and cardiac dysfunction after myocardial infarction through targeting KBTBD7
Source: Cell Death Dis. 2018 Jul 10;9(7):769. doi: 10.1038/s41419-018-0805-5 (PMC6039462; doi:10.1038/s41419-018-0805-5)
Supplement: Supplementary file 1 — Supplementary Figure andTable [file 41419_2018_805_MOESM1_ESM.docx]

**Supplementary Material**


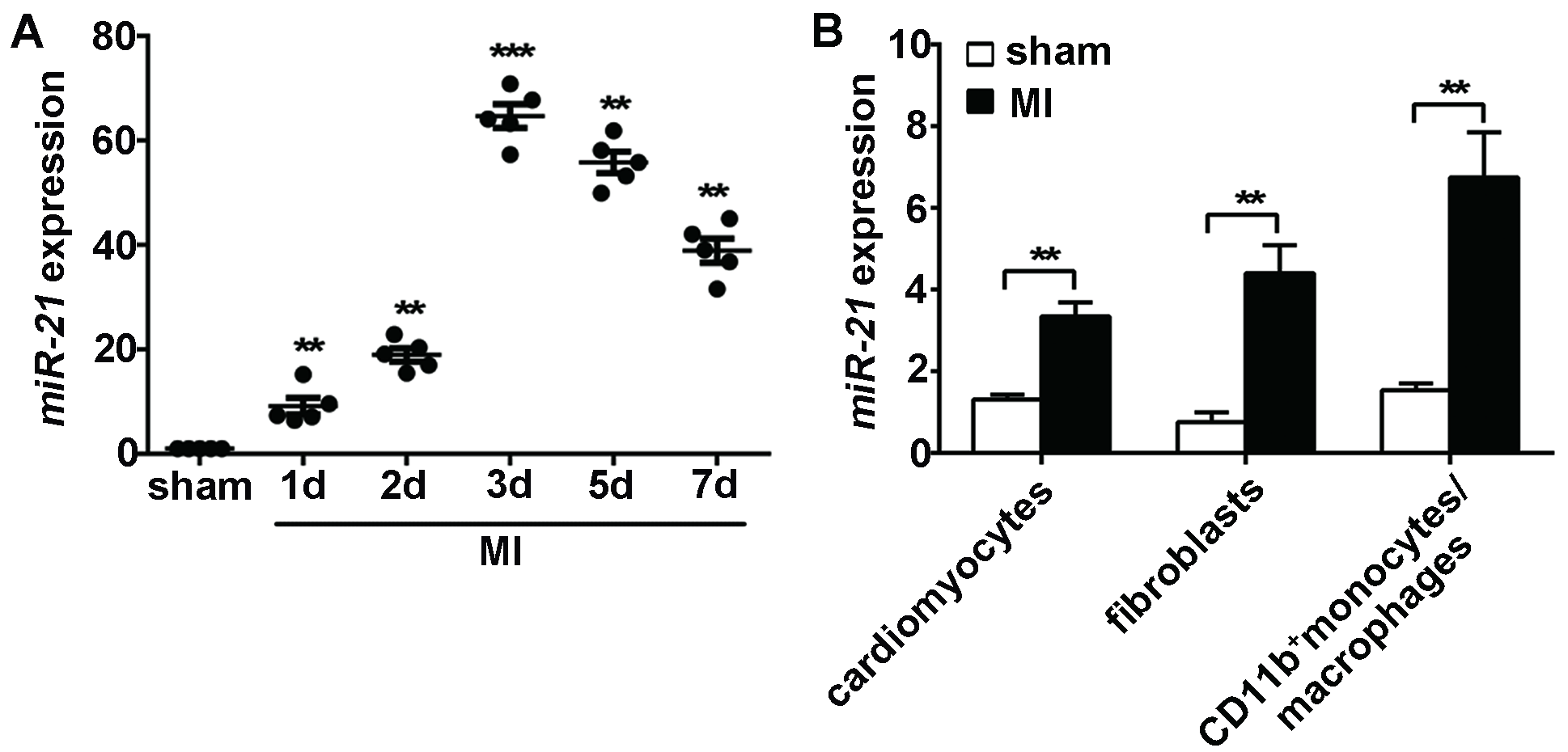


**Figure S1.** **MiR-21 expression is increased** **in the infarcted heart tissue and CD11b^+^ monocytes/macrophages.** (A) Q-PCR analysis of miR-21 expression in heart tissue from sham group or mice at the indicated days after MI. (B) Q-PCR analysis of miR-21 expression in cardiomyocytes, fibroblasts and CD11b^+^ monocytes/macrophages isolated from heart tissue of sham group or mice 1 day after MI. Expression levels were presented as relative fold values compared to sham group (determined as 1) after normalization to *U6*. Data represent the mean ± SEM (n = 5 mice per group). **P* < 0.05, ***P* < 0.01,****P* < 0.001 vs. sham (two-way ANOVA).





**Figure S2. MiR-21 expression is not detected in** **heart tissue from miR-21-deficient mice.** Q-PCR analysis of miR-21 exression (normalization to *U6*) in heart tissue from miR-21-deficient or WT mice. n = 5 mice per group. ND, non-detected.


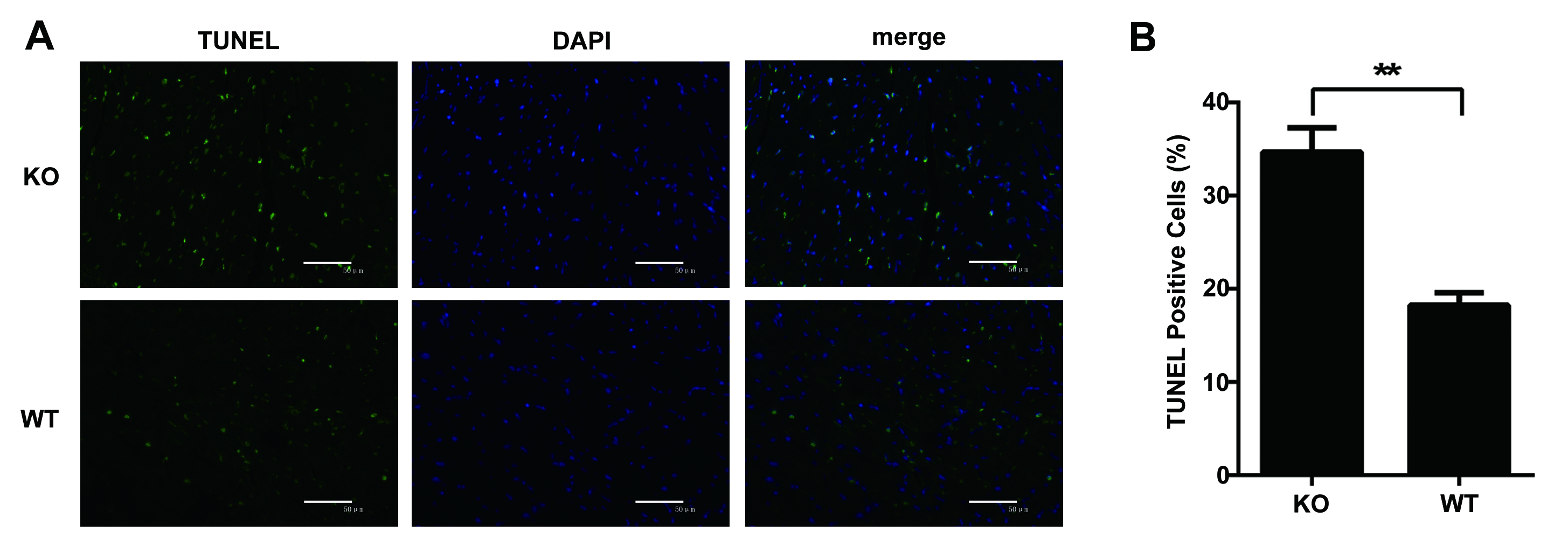


**Figure S3. MiR-21 deficiency increases post-ischemic cardiomyocyte apoptosis.** (A) TUNEL staining (green) of cardiomyocyte apoptosis in the border/infarct area of heart from miR-21 KO or WT mice 1 day after MI. Scale bars = 50 μm. (B) Quantification of TUNEL positive cells was presented as a percentage of cells counted. Data represent the mean ± SEM (n = 5 mice per group) (B). ***P* < 0.01 vs. WT (Student’s t-test).


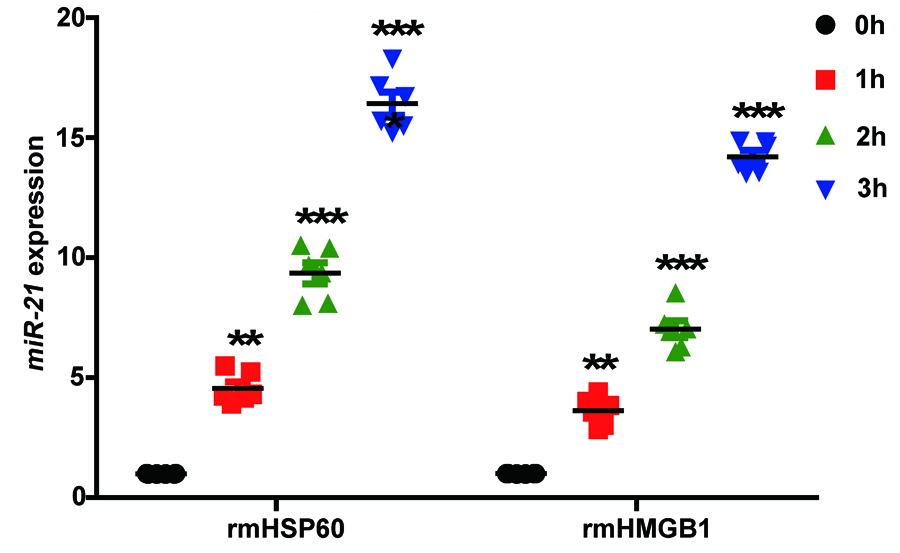


**Figure S4. MiR-21 expression is increased in macrophages treated with DAMPs.** Q-PCR analysis of miR-21 expression in WT macrophages stimulated with rmHMGB1 (1 μg/ml) or rmHSP60 (1 μg/ml) for the indicated times. Expression levels were presented as relative fold values compared to that at 0 h (determined as 1) after normalization to *U6*. Data represent the mean ± SEM (n = 6 independent preparations). ***P* < 0.01, ****P* < 0.001 vs. corresponding 0 h (one-way ANOVA).





**Figure S5. IL-6 production is increased in a dose-dependent manner in macrophages treated with DAMPs.** ELISA of IL-6 production in supernatants of WT macrophages stimulated with the indicated concentrations of rmHMGB1 or rmHSP60 for 8 h. Data represent the mean ± SEM (n = 6 independent preparations). ***P* < 0.01 (one-way ANOVA).





**Figure S6. MiR-21 deficiency promotes DAMP-induced expression of inflammatory cytokine mRNA in macrophages.** Q-PCR analysis of cytokine mRNA expression in miR-21-deficient (KO) or WT macrophages treated with rmHMGB1 (1 μg/ml) (A) or rmHSP60 (1 μg/ml) (B) for the indicated times. Expression levels were presented as relative fold values compared to that in WT at 0 h (determined as 1 for each gene) after normalization to β-actin. **P* < 0.05, ***P* < 0.01 (two-way ANOVA).





**Figure S7. MiR-21 overexpression inhibits DAMP-induced expression of inflammatory cytokine mRNA in macrophages.** (A) Q-PCR analysis of miR-21 expression in macrophages 48 h after transfection with control (ctrl) mimics or miR-21 mimics (normalization to *U6*). (B and C) Q-PCR analysis of cytokine mRNA expression in macrophages 48 h after transfection with miR-21 or control mimics followed by treatment with rmHMGB1 (1 μg/ml) (B) or rmHSP60 (1 μg/ml) (C) for the indicated times. Expression levels were presented as relative fold values compared to that in control mimics at 0 h (determined as 1 for each gene) after normalization to β-actin. ***P* < 0.01, ****P* < 0.001 (two-way ANOVA). Data represent the mean ± SEM (n = 6 independent preparations).

**
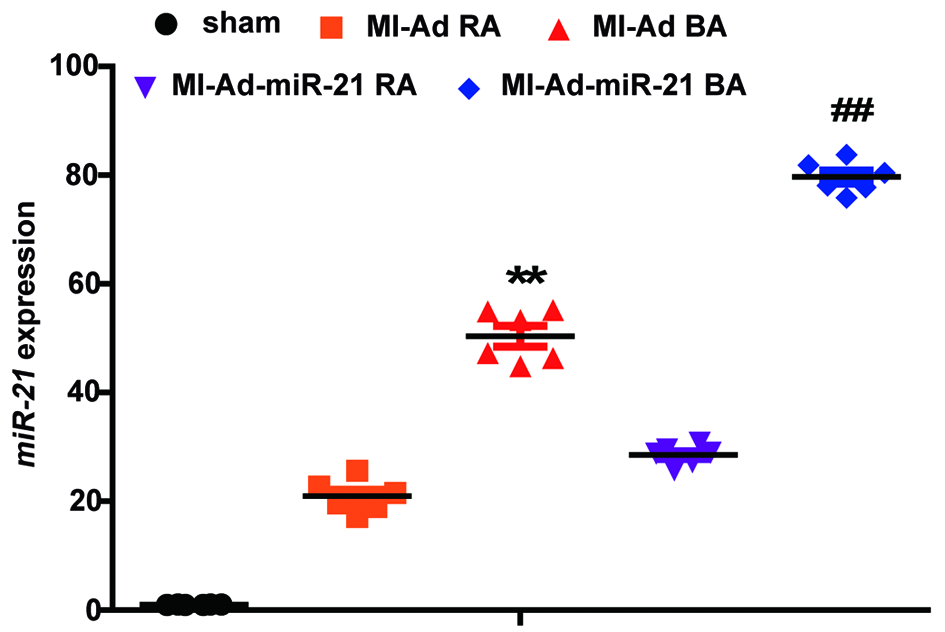
**

**Figure S8. MiR-21 expression is up-regulated in peri-infarct heart tissue after injection with miR-21 adenovirus.** Q-PCR analysis of miR-21 expression in heart tissue of remote area (RA) or border area (BA) from mice 3 days after MI followed by injection with miR-21 (Ad-miR-21) or control adenovirus (Ad) into peri-infarct heart tissue. Expression levels were presented as relative fold values compared to sham group (determined as 1) after normalization to *U6*. Data represent the mean ± SEM (n = 6 mice per group). ***P* < 0.01 vs. MI-Ad RA, ^##^*P* < 0.01 vs. MI-Ad BA (two-way ANOVA).





**Figure S9.** **Knockdown of KBTBD7 suppresses** **DAMP-induced expression of inflammatory cytokine mRNA in macrophages.** Q-PCR analysis of cytokine mRNA expression in macrophages 48 h after transfection with control siRNA or *Kbtbd7* siRNA followed by treatment with rmHMGB1 (1 μg/ml) (A) or rmHSP60 (1 μg/ml) (B) for the indicated times. Expression levels were presented as relative fold values compared to that in control siRNA at 0 h (determined as 1 for each gene) after normalization to β-actin. Data represent the mean ± SEM (n = 6 independent preparations). ***P* < 0.01 vs. ctrl siRNA at the corresponding indicated times (two-way ANOVA).





**Figure S10.** **MiR-21 deficiency aggravates cardiac dysfunction post-MI.** Mice were subjected to MI or sham operation, or KO mice received MI were further injected with adenovirus expressing *Kbtbd7* siRNA (KO-K7 si-MI) or control siRNA (KO-ctrl si-MI) into peri-infarct heart tissue. Cardiac function was measured 2 weeks after MI via echocardiography. Representative M-mode images were shown (n = 10 mice per group).


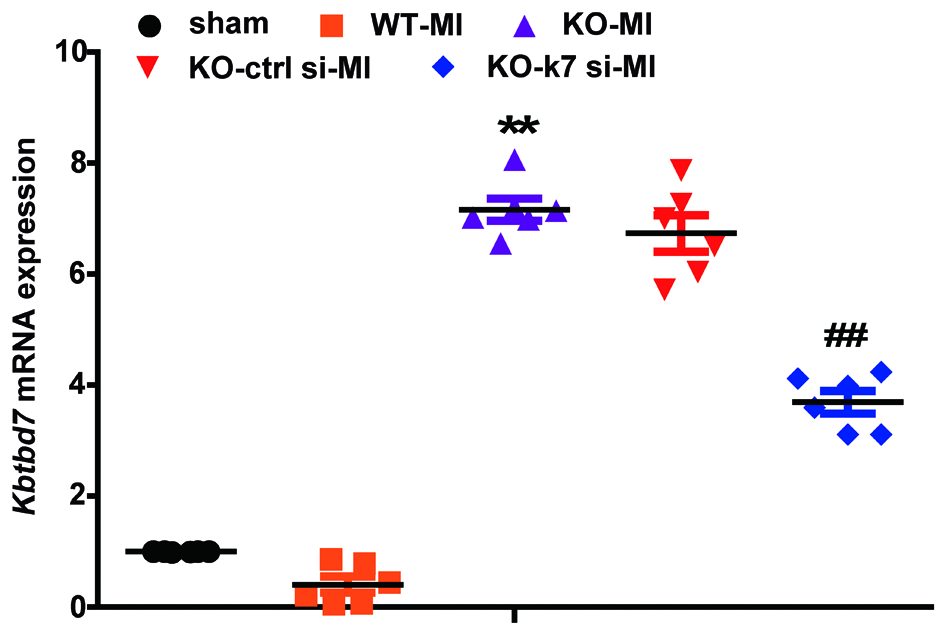


**Figure S11. KBTBD7 expression is down-regulated in peri-infarct heart tissue of miR-21 KO mice after injection with adenovirus expressing Kbtbd7 siRNA.** MiR-21 KO and WT mice were subjected to MI or sham operation, or miR-21 KO mice received MI were further injected with adenovirus expressing *Kbtbd7* siRNA (KO-K7 si-MI) or control siRNA (KO-ctrl si-MI) into peri-infarct heart tissue. Q-PCR analysis of *Kbtbd7* mRNA expression (normalization to GAPDH) in border area of heart tissue from mice. Data represent the mean ± SEM (n = 6 mice per group). ***P* < 0.01 vs. WT-MI; ^##^*P* < 0.01 vs. KO-ctrl si-MI (two-way ANOVA).


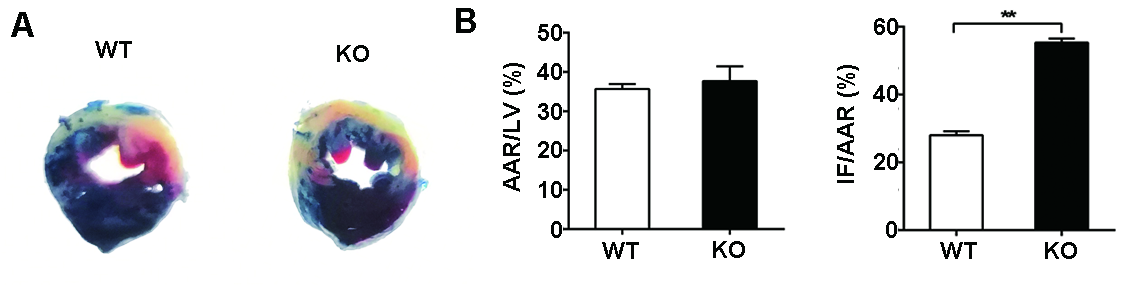


**Figure S12. MiR-21 deficiency increases infarct size post-MI.** (A) Representative images of Evans blue/triphenyl tetrazolium chloride staining of heart sections from miR-21 KO and WT mice 3 days after MI. Blue means the non-risk area of heart, and the remaining is the risk area, which appears red if viable and white if infarcted. (B) The quantitative data for area at risk (AAR) and infarct size (IF) in heart sections from miR-21 KO and WT mice 3 days after MI. LV, left ventricle. Data represent the mean ± SEM (n = 5 mice per group). ***P* < 0.01 vs. WT.

**Table S1.** Q-PCR primers for detection of miR-21 or gene mRNA expression.

|  | Forward | Reverse |
| --- | --- | --- |
| miR-21 | 5′-CGGCTAGCTTATCAGACTGA-3′ | 5′-GTGCAGGGTCCGAGGT-3′ |
| *U6* | 5′- CTCGCTTCGGCAGCACA-3′ | 5′- AACGCTTCACGAATTTGCGT-3′ |
| *Kbtbd7* | 5′-AGCTCAGTTGTATCGGTAGCA-3′ | 5′- CCGGAATAAGGGTCGTAACAGA-3′ |
| *Il1b* | 5′-GGTGTGTGACGTTCCCATTAGAC-3′ | 5′-CATGGAGAATATCACTTGTTGGTTGA-3′ |
| *Il6* | 5′-TAGTCCTTCCTACCCCAATTTCC-3′ | 5′-TTGGTCCTTAGCCACTCCTTC-3′ |
| *Tnf* | 5′- AAGCCTGTAGCCCACGTCGTA-3′ | 5′-GGCACCACTAGTTGGTTGTCTTTG-3′ |
| *Gapdh* | 5′- AGGTCGGTGTGAACGGATTTG-3′ | 5′-TGTAGACCATGTAGTTGAGGTCA-3′ |
